# CAFE: a software suite for analysis of paired-sample transposon insertion sequencing data

**DOI:** 10.1093/bioinformatics/btaa1086

**Published:** 2021-01-04

**Authors:** Anna Abramova, Adriana Osińska, Haveela Kunche, Emil Burman, Johan Bengtsson-Palme

**Affiliations:** Department of Infectious Diseases, Institute of Biomedicine, The Sahlgrenska Academy, University of Gothenburg, Guldhedsgatan 10A, SE-413 46 Gothenburg, Sweden; Centre for Antibiotic Resistance Research (CARe) at University of Gothenburg, SE- 40530 Gothenburg, Sweden; Department of Infectious Diseases, Institute of Biomedicine, The Sahlgrenska Academy, University of Gothenburg, Guldhedsgatan 10A, SE-413 46 Gothenburg, Sweden; Centre for Antibiotic Resistance Research (CARe) at University of Gothenburg, SE- 40530 Gothenburg, Sweden; Department of Water Protection Engineering and Environmental Microbiology, Faculty of Geoengineering, University of Warmia and Mazury in Olsztyn, 10-720 Olsztyn, Poland; Department of Infectious Diseases, Institute of Biomedicine, The Sahlgrenska Academy, University of Gothenburg, Guldhedsgatan 10A, SE-413 46 Gothenburg, Sweden; Centre for Antibiotic Resistance Research (CARe) at University of Gothenburg, SE- 40530 Gothenburg, Sweden; Programme in Infection Biology, School of Bioscience, University of Skövde, 541 28 Skövde, Sweden; Department of Infectious Diseases, Institute of Biomedicine, The Sahlgrenska Academy, University of Gothenburg, Guldhedsgatan 10A, SE-413 46 Gothenburg, Sweden; Centre for Antibiotic Resistance Research (CARe) at University of Gothenburg, SE- 40530 Gothenburg, Sweden; Department of Infectious Diseases, Institute of Biomedicine, The Sahlgrenska Academy, University of Gothenburg, Guldhedsgatan 10A, SE-413 46 Gothenburg, Sweden; Centre for Antibiotic Resistance Research (CARe) at University of Gothenburg, SE- 40530 Gothenburg, Sweden

## Abstract

**Summary:**

Sequencing of transposon insertion libraries is used to determine the relative fitness of individual mutants at a large scale. However, there is a lack of tools for specifically analyzing data from such experiments with paired sample designs. Here, we introduce CAFE—Coefficient-based Analysis of Fitness by read Enrichment—a software package that can analyze data from paired transposon mutant sequencing experiments, generate fitness coefficients for each gene and condition and perform appropriate statistical testing on these fitness coefficients.

**Availability and implementation:**

CAFE is implemented in Perl and R. The source code is freely available for download under the MIT License from https://github.com/bengtssonpalme/cafe and http://microbiology.se/software/cafe/

**Supplementary information:**

Supplementary data are available at *Bioinformatics* online.

## 1 Introduction

Over the last years, a variety of approaches for investigating the fitness of mutants at a large scale have emerged (Chao *et al.*, 2016; Goodman *et al.*, 2011; van Opijnen and Camilli, 2013). Generally, these approaches are based on insertion of a transposase *en masse* in the target genome, followed by sequencing of tags from these mutants that allow determination of their relative fitness in experimental or *in vivo* conditions. While a number of software packages exist for analysis of this type of data (Blanchard *et al.*, 2015; McCoy *et al.*, 2017; Zhao *et al.*, 2017; Zomer *et al.*, 2012), these packages lack a central feature that is critical for specifically addressing the fitness effects of specific genes that are unique for a given condition. The missing feature is the ability to compare a given treatment condition to a control, under the assumption that the initial starting collection of transposon mutants come from the same pool for each paired replicate. This type of experimental setup allows a direct assessment of the genes that have significant effects on fitness specifically when one experimental factor is altered (such as exposure to a selective agent, the presence of other species etc.). In this paper, we introduce CAFE—Coefficient-based Analysis of Fitness by read Enrichment—a software package to analyze data from paired transposon mutant sequencing experiments, generate fitness coefficients for each gene and condition, as well as perform appropriate statistical testing on these fitness coefficients.

## 2 Implementation

The CAFE package is based upon the concept of condition-specific fitness coefficients (FCs). Each FC describes the relative importance of a gene under a certain condition and is derived from a comparison of how mutants coming from the same source population differ after growth in a studied condition and a control condition. The FC is defined as:

(1) $$\text{FC}=-\text{log}\;2\;(\frac{n_{\text{cond}}/s_{\text{cond}}}{n_{\text{ctrl}}{/s}_{\text{ctrl}}}\;/\frac{u_{\text{cond}}/s_{\text{cond}}}{u_{\text{ctrl}}{/s}_{\text{ctrl}}})$$

where *FC* is the condition-specific FC, *n* is the number of read counts assigned to a gene, *s* the total number of mapped reads from a sample, *u* the number of reads mapped to intergenic regions, and ‘cond’ and ‘ctrl’ represent the testing condition and the control, respectively. The reads corresponding to intergenic transposon insertions *u* are used as a normalization factor assumed to show no effect between the condition and the control, but can be set to some other factor if a better no-effect control exists.

CAFE is a set of command-line tools for analysis of sequence data implemented in Perl, combined with an R package for statistical analysis of the read counts generated. The entire software package should be functional under any version of Unix or Linux, including MacOS. The R package also runs well in the Windows version of R. The command-line tools are dependent on cutadapt (Martin, 2011), TrimGalore! (https://www.bioinformatics.babraham.ac.uk/projects/trim_galore/) and Bowtie2 (Langmead and Salzberg, 2012) for full functionality.

## 3 Evaluation

To assess the performance of the CAFE package compared to other commonly used software for transposon sequencing analysis, we used data from an InSeq experiment comparing *Pseudomonas aeruginosa* transposon mutant libraries after overnight growth to the frozen state of the same libraries (available as CAFE example data from https://microbiology.se/sw/cafe/example_data.tgz). We analyzed this data using CAFE and then ran ESSENTIALS (Zomer *et al.*, 2012), MAGenTA (McCoy *et al.*, 2017) and TnseqDiff (Zhao *et al.*, 2017) on either the reads from the experiment or the counts resulting from the Perl portion of CAFE, depending on the input required by the different analysis tools (Table 1; see Supplementary Text for details). A few important conclusions can be drawn from this analysis. First, ESSENTIALS and to some degree also TnseqDiff produce unrealistically small *P*-values (Supplementary Fig. S1). For example, ESSENTIALS produces *P*-values smaller than 10^−100^ and TnseqDiff generates *P*-values as small as 10^−30^ for this dataset. With only five replicates in each group and considerable within-treatment variation, such small *P*-values hints at an over-confident statistical method. Furthermore, MAGenTA indicates that virtually all genes have significant differences (Supplementary Fig. S2), which is very unlikely to be true, and in any case is not a particularly useful result in terms of filtering out relevant hits. We also see that all four tools agree on that 689 genes have significant differences between the two treatments (Supplementary Fig. S3). MAGenTA stands out as the most liberal, having identified 1691 genes as significant that were not reported by any of the other tools. Furthermore, it is notable that ESSENTIALS share 879 reported genes with only MAGenTA (which reports almost all genes as significant), while CAFE and TnseqDiff only share 332 and 16 reported genes with MAGenTA, respectively. It is worth pointing out that we have no way of knowing the ‘true’ result in this case—we can only make reasonable assumptions on what a plausible distribution of significantly differential genes would look like, and the results reported by CAFE and ESSENTIALS seem to match the expected distributions best. We also investigated the robustness against false positives on a no-effect dataset and found that CAFE and ESSENTIALS far outperformed the other two tools in this respect (Supplementary Text and Supplementary S4 and S5).

**Table 1.** Comparison of CAFE with three other commonly used software for transposon sequencing analysis

	CAFE	ESSENTIALS	MAGenTA	TnseqDiff
Total number of reported genes	5703	5697	5697	5700
Number of genes with adjusted *P*-value ≤ 0.05	2375	2847	4920	973
Percentage significant genes	41.6%	50.0%	86.4%	17.1%

## 4 Conclusions

Our evaluation of currently used statistical methods for analysis of transposon insertion sequencing data reveals substantial flaws in the methodological assumptions, particularly when the samples are paired. We here introduce a new solution to this paired-sample transposon sequencing library problem in the form of a software package—CAFE—which is capable of performing the bioinformatic processing of sequence data from such experiments, as well as performing statistical analysis. The R package part of CAFE can operate on any type of count data from paired transposon sequencing experiments, regardless of if the CAFE tools were used for preprocessing or not. The CAFE package is open source and available from GitHub (https://github.com/bengtssonpalme/cafe) as well as from https://microbiology.se/software/cafe/

## Funding

This work was supported by the Swedish Research Council for Environment, Agricultural Sciences and Spatial Planning (FORMAS; grant 2016-00768), the Sahlgrenska Academy at the University of Gothenburg, the Swedish Research Council (VR; grant 2019-00299) under the frame of JPI AMR (EMBARK; JPIAMR2019-109), the Centre for Antibiotic Resistance Research at the University of Gothenburg, the Adlerbertska research foundation, the O.E. and Edla Johansson foundation, the Swedish Cancer and Allergy fund (Cancer- och Allergifonden) and Längmanska Kulturfonden.

*Conflict of Interest*: none declared.

## Supplementary Material

btaa1086_Supplementary_Data
