# Supplementary material for: CAFE: a software suite for analysis of paired-sample transposon insertion sequencing data
Source: Bioinformatics. 2021 Jan 4;37(1):121–2. doi: 10.1093/bioinformatics/btaa1086 (PMC8034522; doi:10.1093/bioinformatics/btaa1086)
Supplement: btaa1086_Supplementary_Data [file btaa1086_supplementary_data.zip › Supplementary_Methods_and_Results Revised.pdf]

CAFE: A software suite for analysis of paired-sample transposon insertion sequencing data

Anna Abramova, Adriana Osińska, Haveela Kunche, Emil Burman, Johan Bengtsson-Palme

Supplementary Methods

We compared the performance of the CAFE package to a number of available tools for transposon sequencing analysis, including ESSENTIALS (Zomer *et al.*, 2012), MAGenTA (McCoy *et al.*, 2017) and TnseqDiff (Zhao *et al.*, 2017). We also considered a comparison to the PIMMS package (Blanchard *et al.*, 2015), but decided against this comparison as it does not handle experimental setups with replicates in a way that was compatible with our data. For the comparison, we used the same dataset produced by sequencing an INSeq (Goodman *et al.*, 2011) library of *Pseudomonas aeruginosa* directly from the freezer and after overnight growth at 28°C.

Sequencing reads were preprocessed by running them through the CAFE package, using the following options:

```
cafe-map -i all.fastq.gz -o all/ -b metadata.txt -r PAO1 --cpu 32
```

where all.fastq.gz is the input file containing all replicate experiments (from one sequencing run), metadata.txt contains the library and barcode information and PAO1 is a Bowtie2 (Langmead and Salzberg, 2012) database built from the *Pseudomonas aeruginosa* PAO1 genome, downloaded from https://www.pseudomonas.com/downloads/pseudomonas/pgd_r_20_2/Pseudomonas_aeruginosa_PAO1_107/Pseudomonas_aeruginosa_PAO1_107.fna.gz (Winsor *et al.*, 2016). Using this command, the data was split by barcodes and all non-genomic DNA was removed. Reads were quality trimmed using TrimGalore! (Babraham Bioinformatics, 2012) as built into the CAFE package.

1. CAFE:

For the CAFE package evaluation, the sequencing reads for each individual library were first mapped with cafe-map.pl with default parameters to the *P. aeruginosa* genome PAO1:

```
cafe-map -T -S -i Pa_ON_1.fq -o all/ -b metadata.txt -r PAO1 --cpu 32
```

The -T and -S options skip the read trimming and sorting by barcode, respectively, as that was done in the previous step. The resulting SAM files were annotated with cafe-annotate, producing an output file with read counts for each of the samples (cafe_combined_out.annot.txt):

```
cafe-annotate Pseudomonas_aeruginosa_PAO1_107.txt Pa_ON_1.fq.sam Pa_ON_2.fq.sam
Pa_ON_3.fq.sam Pa_ON_4.fq.sam Pa_ON_5.fq.sam Pa_freezer_1.fq.sam
Pa_freezer_2.fq.sam Pa_freezer_3.fq.sam Pa_freezer_4.fq.sam Pa_freezer_5.fq.sam
```

The output table was imported into R and in the next step, the fitCof function with default parameters was used to calculate fitness coefficients between the two conditions. The significant effect of a gene on fitness was estimated by performing a t-test (default function in CAFE):

```
pa_fc <- fitCof("Pa_ON", "Pa_freezer")
pa_rank <- rankByP(pa_fc)
write.table(pa_rank, file = "cafe_pa_rank_corr.csv", quote = FALSE, sep = "\t")
```

2. MAGenTA:
Sequencing reads were mapped to the *P. aeruginosa* PAO1 reference genome using Bowtie 1 with default package specific parameters:

```
bowtie -n1 --best -y -m1 PAO1 Pa_ON_1.fq > Pa_ON_1.map
```

For each paired library, fitness coefficient was calculated using calc_fitness.py providing -uncol UNCOL for the uncollapsed reads and -b BOTTLEALL for the calculation of bottleneck value used for normalization:

```
python calc_fitness.py -uncol UNCOL -b BOTTLEALL -ref
GCF_000006765.1_ASM676v1_genomic.gbff -t1 Pa_freezer_1.map -t2 Pa_ON_1.map -out
Pa_L1.csv
```

Fitness for individual insertions was then aggregated by gene using aggregate.pl:

```
python aggregate.py -o Pa_aggregate_fitness.csv -w WEIGHTED -c
GCF_000006765.1_ASM676v1_genomic.gbff Pa_L1.csv Pa_L2.csv Pa_L3.csv Pa_L4.csv
Pa_L5.csv
```

The original scripts provided within MAGenTA package do not allow statistical comparisons between two time points within the same experiment. To achieve accommodate for such analyses, we modified the compGenes.pl to perform one sample t-test (Supplementary Code 1).

We also performed p-values Benjamini-Hochberg correction using p.adjust:

```
padj_res <- p.adjust(pval, method= "BH")
```

3. TnseqDiff:
TnseqDiff requires counts as input. The table of counts (cafe_combined_out.txt) generated by the CAFE package tool cafe-annotate was used as input for the TnseqDiff function with default parameters:

```
TnseqDiff(countData, geneID, pool, condition, weights="equal", bayes=FALSE,
p.nb=FALSE, norm=TRUE, cut=0)
```

4. ESSENTIALS:
We used the ESSENTIALS web interface (http://bamics2.cmbi.ru.nl/websoftware/essentials/) to perform the data analysis. Sequencing reads were first mapped to the *P. aeruginosa* PAO1 annotated genome and the tool was run using default settings.

We also performed all of the above analyses on the Pa_ON libraries split in half, to generate a dataset with (in theory) no effects on any genes but with maintained variance structure. We will refer to this dataset as the 'no-effect' dataset below.

Supplementary Results and Discussion

It is notable that ESSENTIALS, MAGenTA and TnseqDiff all produce unrealistically small p-values on our testing data (Fig. S1). On the more extreme end, ESSENTIALS produces p-values smaller than 10^{-100}, while TnseqDiff and MAGenTA generate p-values as small as 10^{-30} and 10^{-15}, respectively, for this dataset. Since there is substantial variation in the experimental data and there are only five replicates per treatment, this level of perceived confidence can be very misleading to the user. To further explore the source of this confidence, we next examined the performance of the four tools on only one of the two treatment conditions, but with each replicate samples split in two. Thus, all comparisons should show no significant differences (as they are drawn from the same population for mutant frequencies), while we still retain the variance structure of the data. This additional analysis revealed that even in a case where populations from the same treatment were compared, MAGenTA indicated the majority of genes to be significantly affected (Fig. S4). Thus, we conclude that MAGenTA makes unreasonable assumptions on the data and may not be suitable for statistical analysis of paired data sets. Furthermore, even on this data where there should be virtually no differences between the two compared groups, TnseqDiff indicates that several genes are significantly affected, giving p-values as small as 10^{-15} after correction for multiple testing. Notably, CAFE and ESSENTIALS handle this situation as would be expected and return no significant genes after correction for multiple testing (Fig. S5) and the expected evenly distributed uncorrected p-values for this no-effect dataset, particularly in the case of CAFE (Fig. S6).

In both these datasets, MAGenTA indicates that almost all genes have significant differences (Fig. S2 and S5), which is obviously an error in the no-effect data and very unlikely to be true also in the example dataset. Many of these genes are not indicated to be significant by any of the other three tools (Fig. S3). All in all, CAFE seems to be far better at handling datasets with no effects than any of the other tools and also (together with ESSENTIALS) shows the expected distributions of p-values on the testing data set (Fig. S2). It is notable that TnseqDiff returns much fewer significant genes than CAFE on the example dataset, but far more significant genes on the no-effect dataset. This indicates a certain conservativeness against reporting too many significant hits, but at the same time hints at a weakness in controlling for false positives.

We also investigated the effect of disabling the correction factor for uneven mutant distributions across samples (the u parameter in formula 1). On the example dataset, changing this parameter had virtually no effect on the analysis ($R^2 = 0.9993$ on results between the two tests). However, this is expected as this parameter mostly is intended to avoid misleading results due to one dataset having extremely high relative abundances of just one or a few genes. Importantly, this analysis shows that in typical cases, the u parameter can be ignored, but it also has no negative effects on regular analyses.

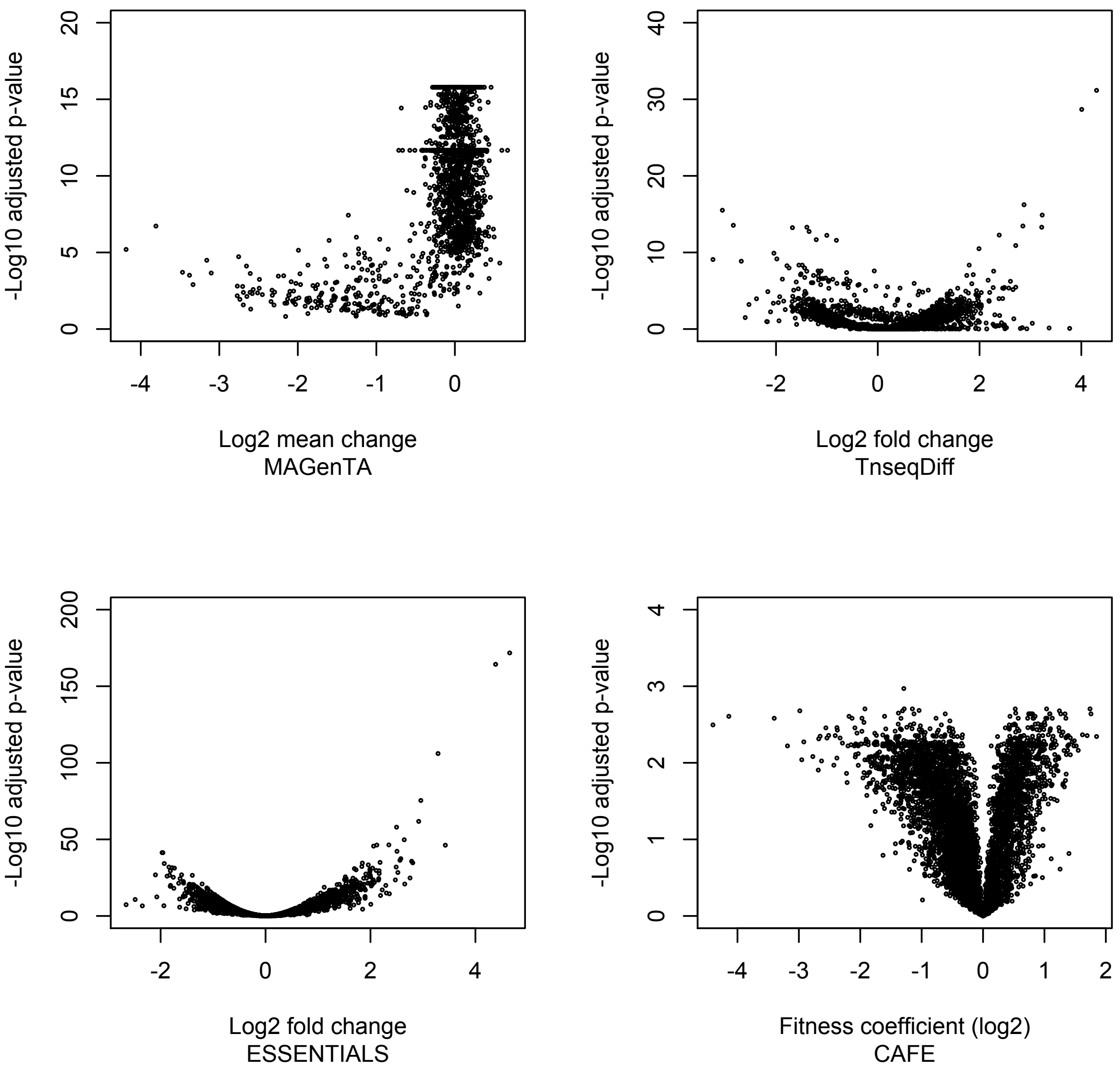

Figure S1. Volcano plots showing the -log10 adjusted p-values and the mean log fold change/fitness coefficients for the four different methods when applied to the example dataset.

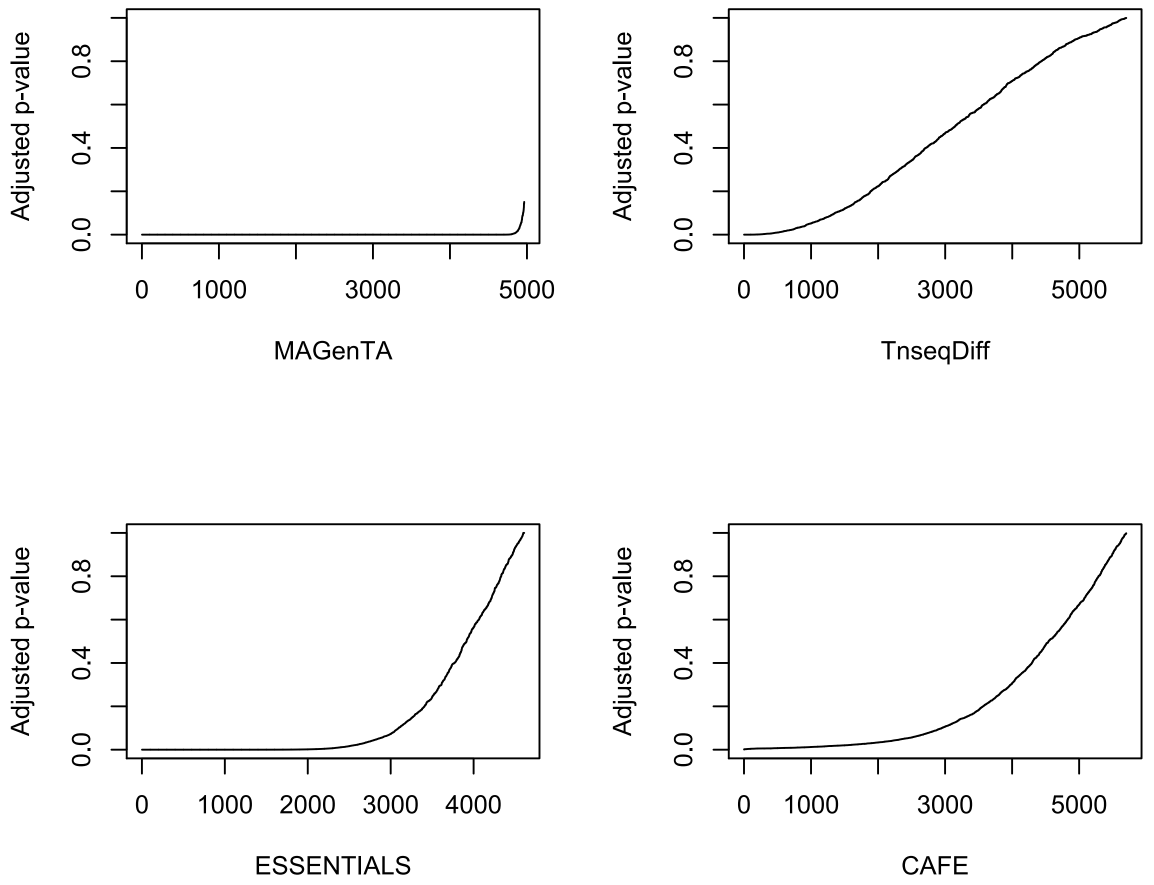

Figure S2. Sorted adjusted p-values for the four methods when applied to the example dataset. The p-values are listed from smallest to largest.

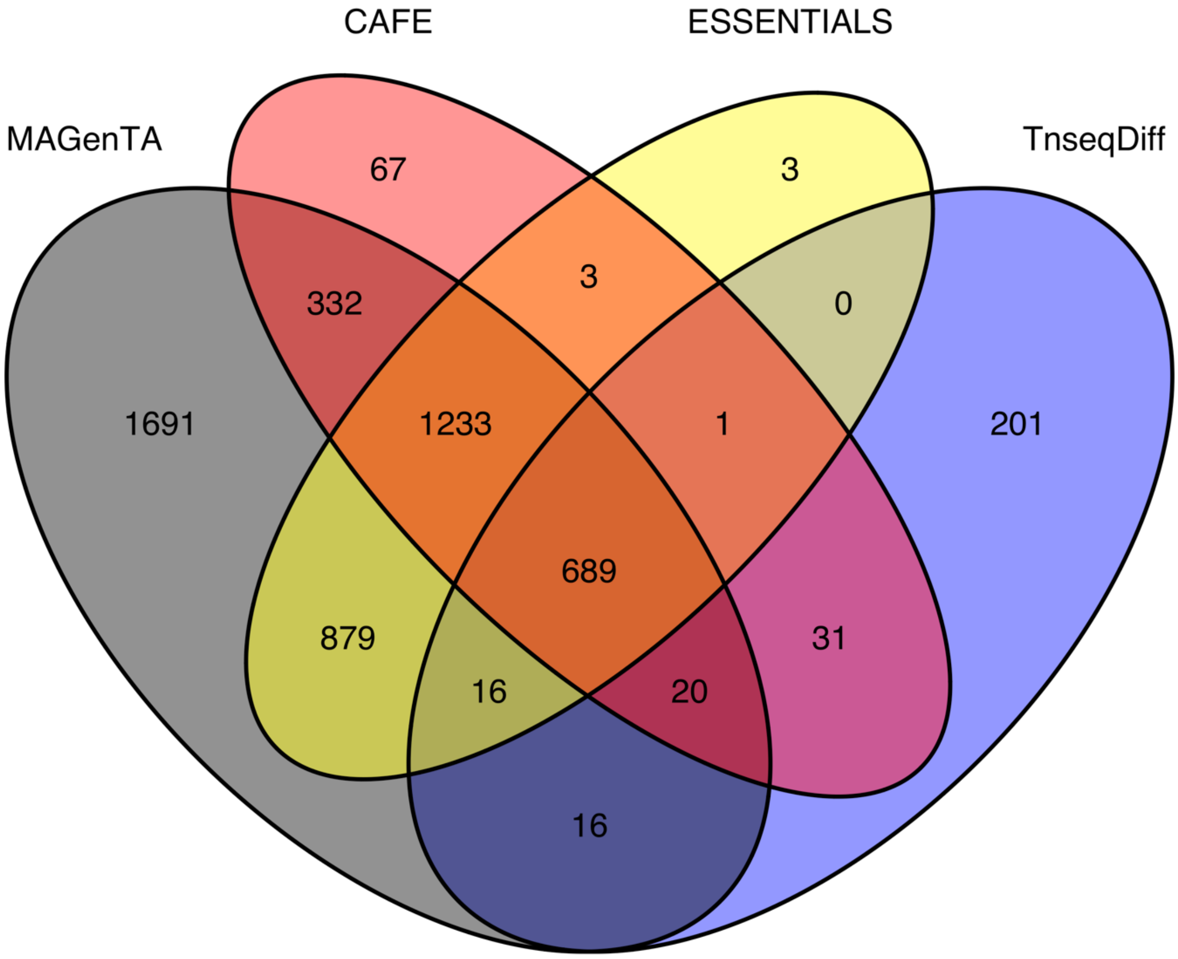

Figure S3. Venn diagram showing the overlap of the genes reported as significant by the four different methods when applied to the example dataset.

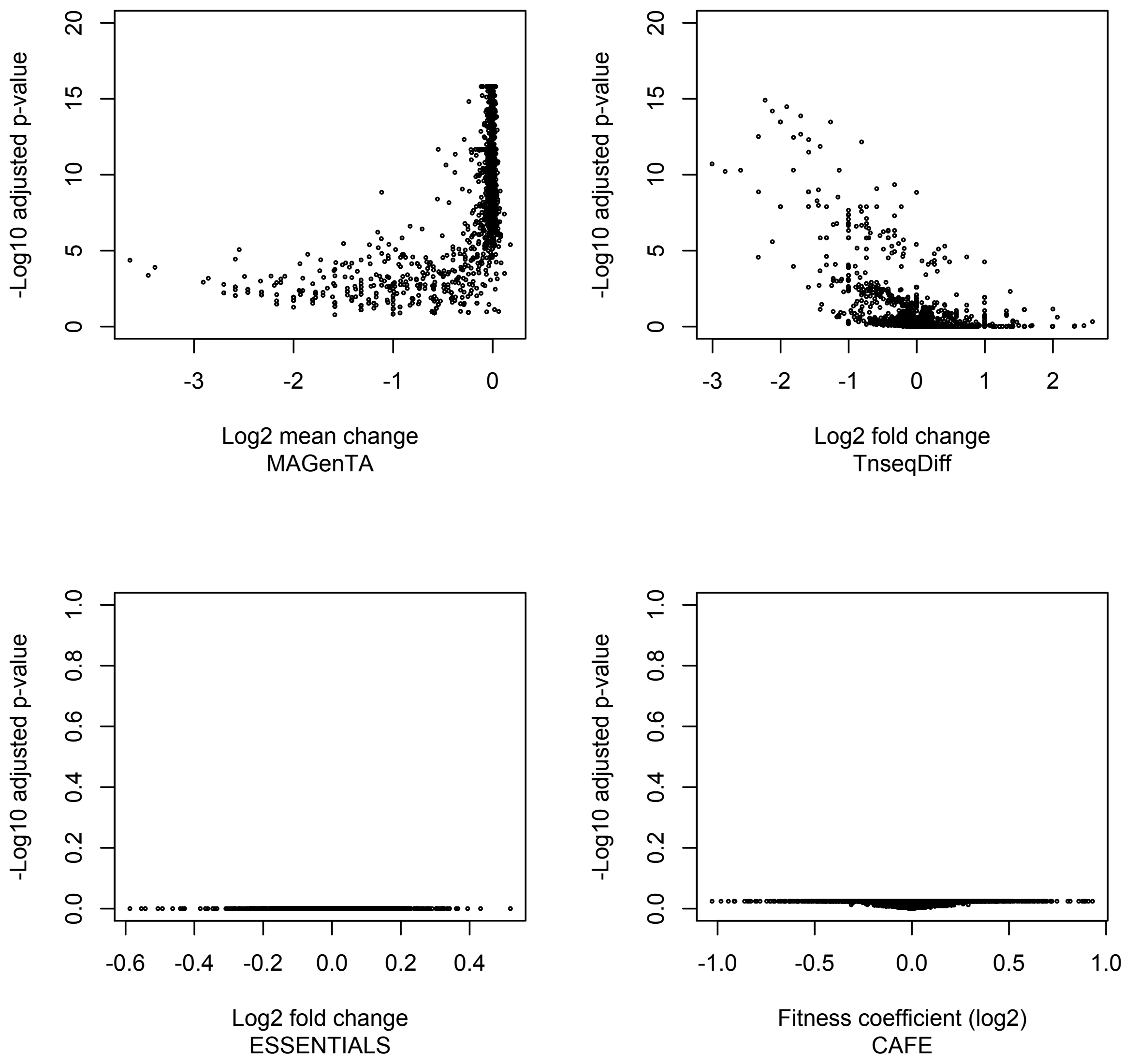

Figure S4. Volcano plots showing the -log10 adjusted p-values and the mean log fold change/fitness coefficients for the four different methods when applied to the no-effect dataset.

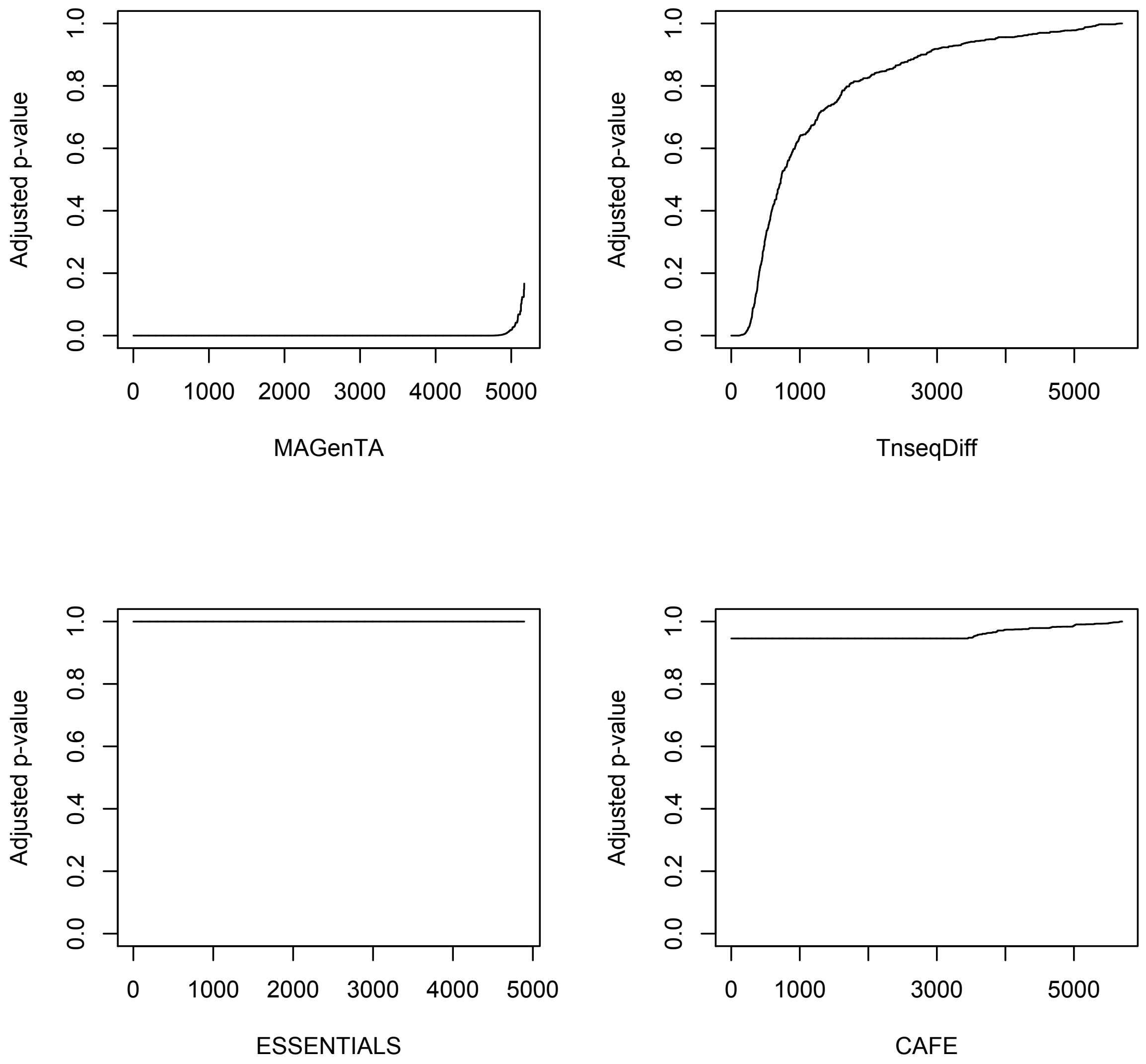

Figure S5. Sorted adjusted p-values for the four methods when applied to the no-effect dataset. The p-values are listed from smallest to largest.

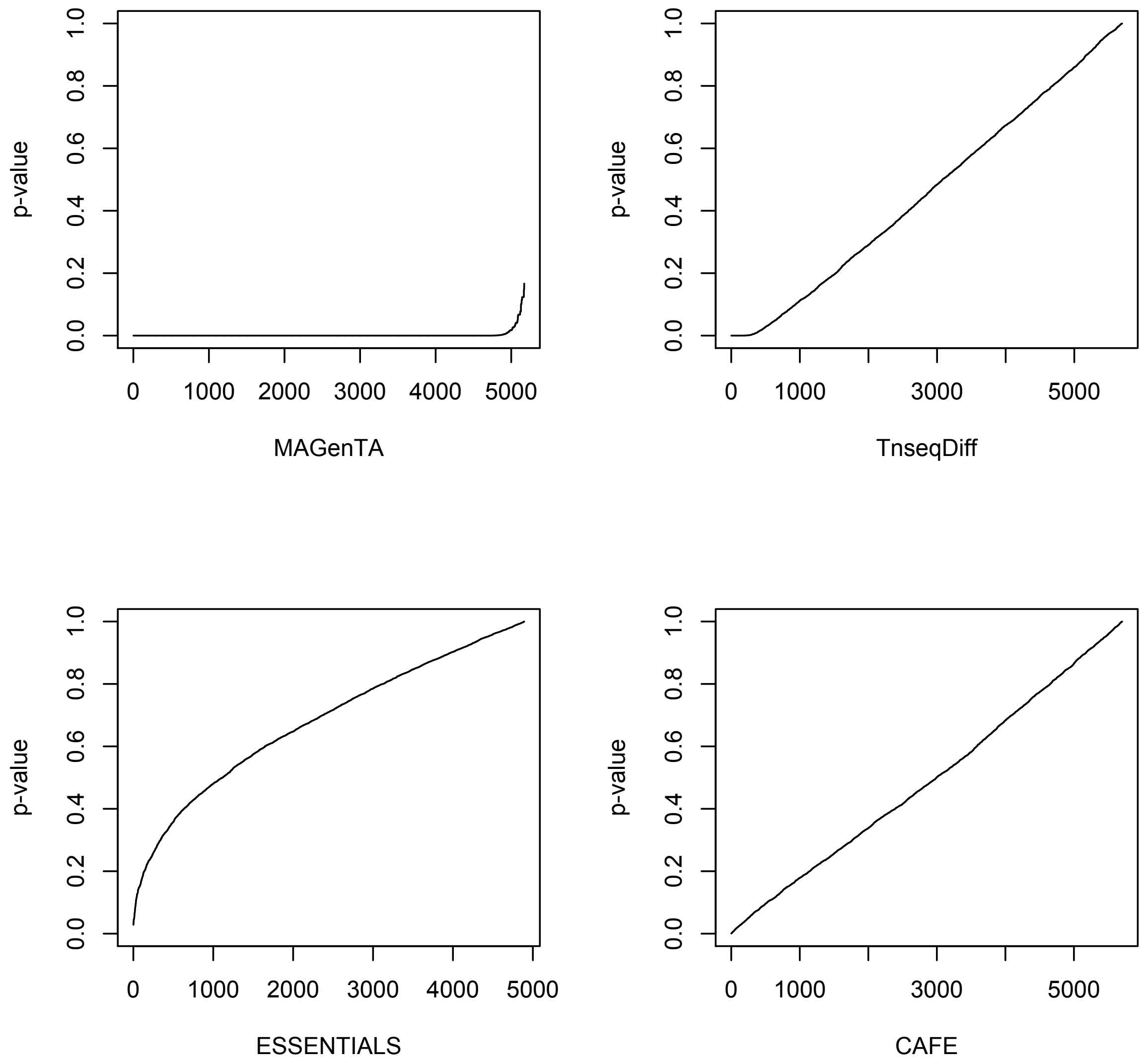

Figure S6. Sorted uncorrected p-values for the four tools when applied to the no-effect dataset. The p-values are listed from smallest to largest, and clearly show the expected linear distribution across the set of genes for CAFE and reasonable trends for TnseqDiff and ESSENTIALS. MAGenTA, as in the previous analysis, indicates that almost all genes have significant differences.

References

Babraham Bioinformatics (2012) Trim Galore! https://www.bioinformatics.babraham.ac.uk/projects/trim_galore/

Blanchard,A.M. *et al.* (2015) Transposon insertion mapping with PIMMS - Pragmatic Insertional Mutation Mapping System. *Frontiers in genetics*, **6**, 139.

Goodman,A.L. *et al.* (2011) Identifying microbial fitness determinants by insertion sequencing using genome-wide transposon mutant libraries. *Nature Protocols*, **6**, 1969–1980.

Langmead,B. and Salzberg,S.L. (2012) Fast gapped-read alignment with Bowtie 2. *Nature Methods*, **9**, 357–359.

McCoy,K.M. *et al.* (2017) MAGenTA: a Galaxy implemented tool for complete Tn-Seq analysis and data visualization. *Bioinformatics (Oxford, England)*, **33**, 2781–2783.

Winsor,G.L. *et al.* (2016) Enhanced annotations and features for comparing thousands of Pseudomonas genomes in the Pseudomonas genome database. *Nucleic Acids Res.*, **44**, D646-653.

Zhao,L. *et al.* (2017) TnseqDiff: identification of conditionally essential genes in transposon sequencing studies. *BMC Bioinformatics*, **18**.

Zomer,A. *et al.* (2012) ESSENTIALS: Software for Rapid Analysis of High Throughput Transposon Insertion Sequencing Data. *PLoS ONE*, **7**, e43012.
