# Supplementary figures and images for: CAFE: a software suite for analysis of paired-sample transposon insertion sequencing data
Source: Bioinformatics. 2021 Jan 4;37(1):121–2. doi: 10.1093/bioinformatics/btaa1086 (PMC8034522; doi:10.1093/bioinformatics/btaa1086)

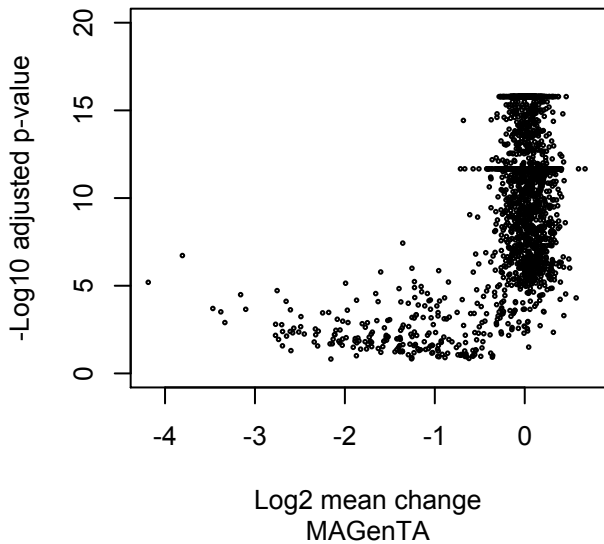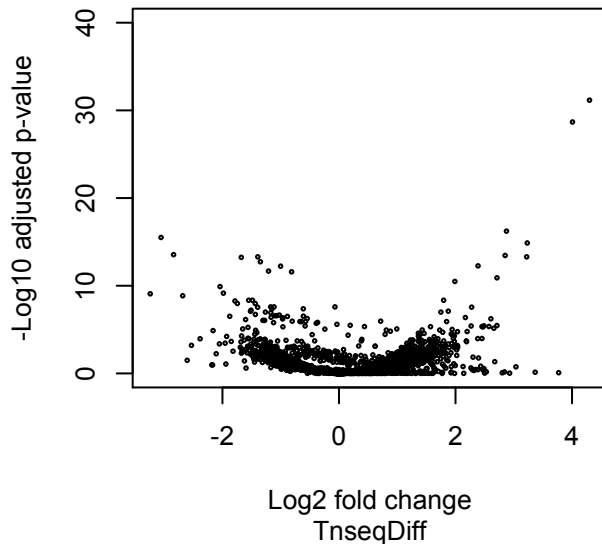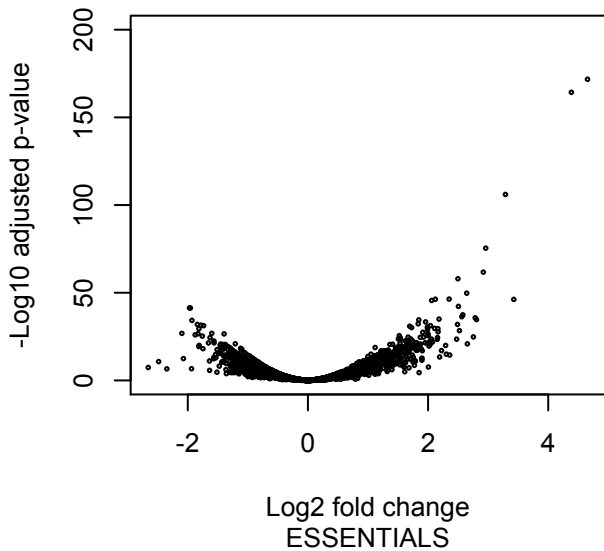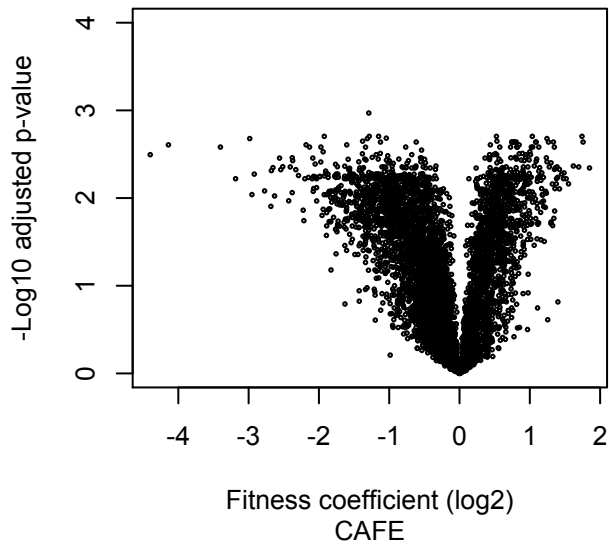

Supplement: btaa1086_Supplementary_Data [file btaa1086_supplementary_data.zip › Fig_S1.pdf]

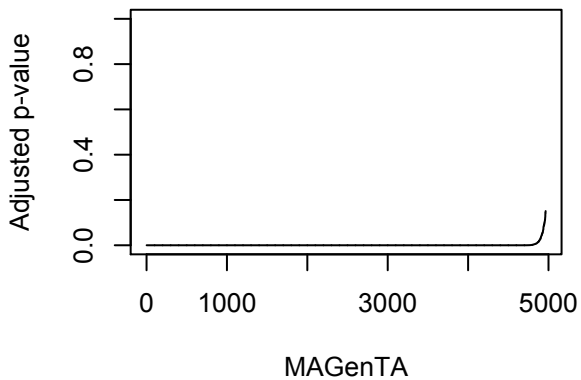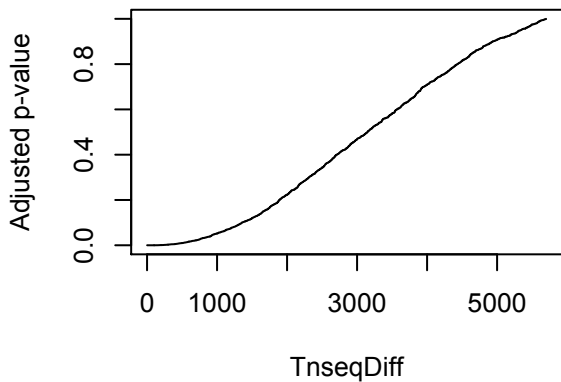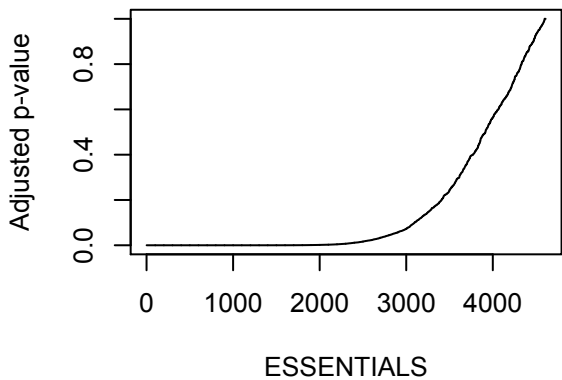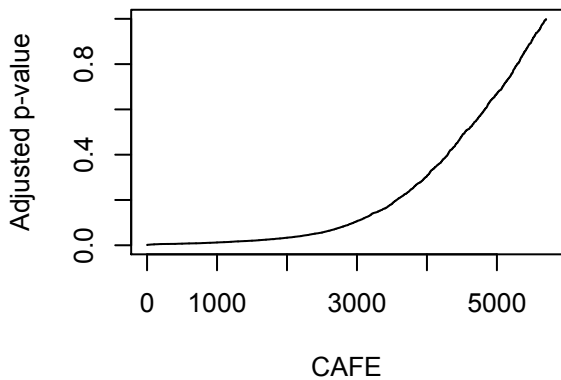

Supplement: btaa1086_Supplementary_Data [file btaa1086_supplementary_data.zip › Fig_S2.pdf]

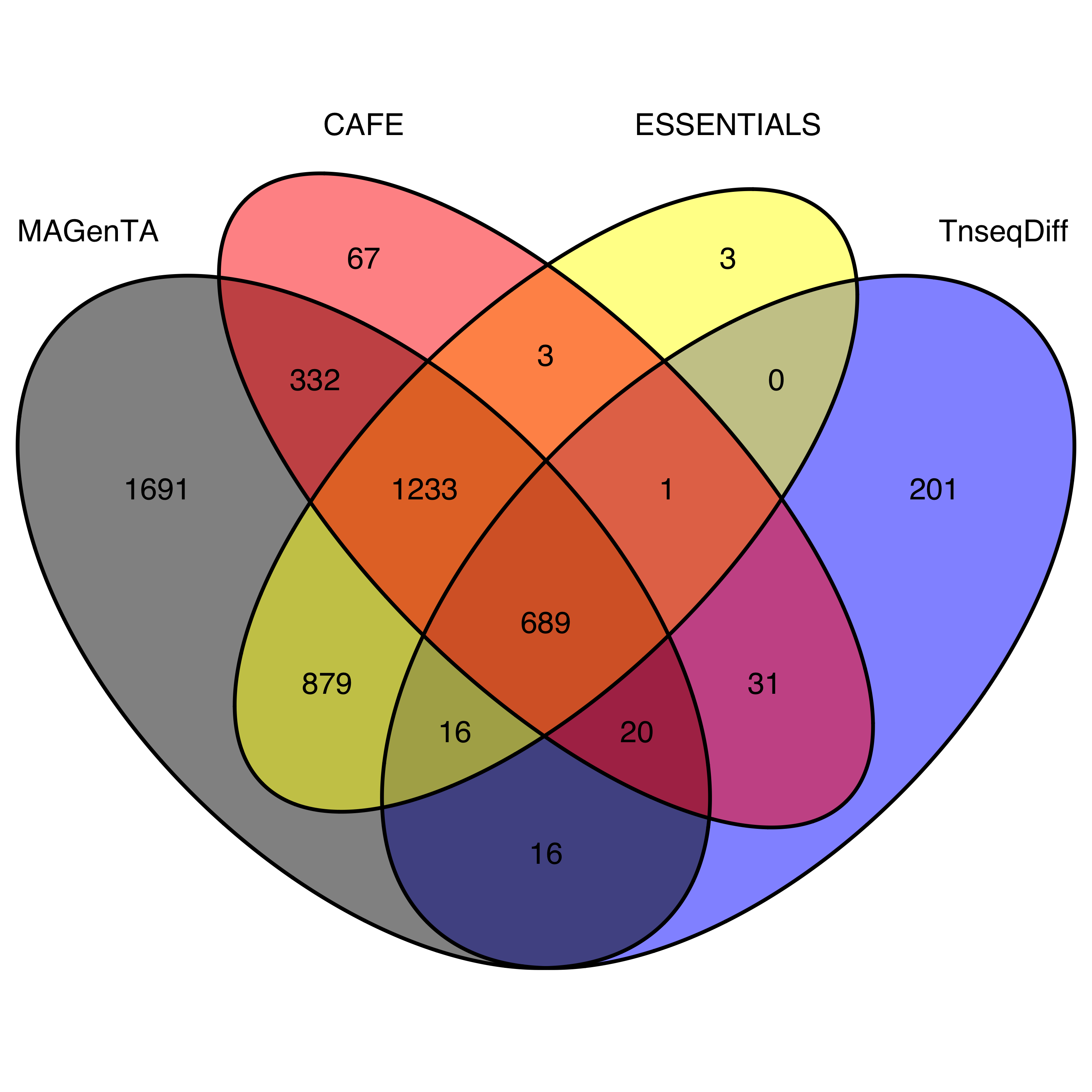

Supplement: btaa1086_Supplementary_Data [file btaa1086_supplementary_data.zip › Fig_S3.png]

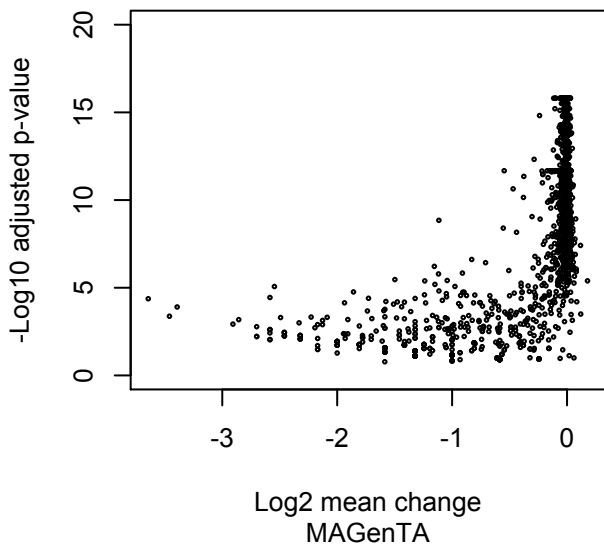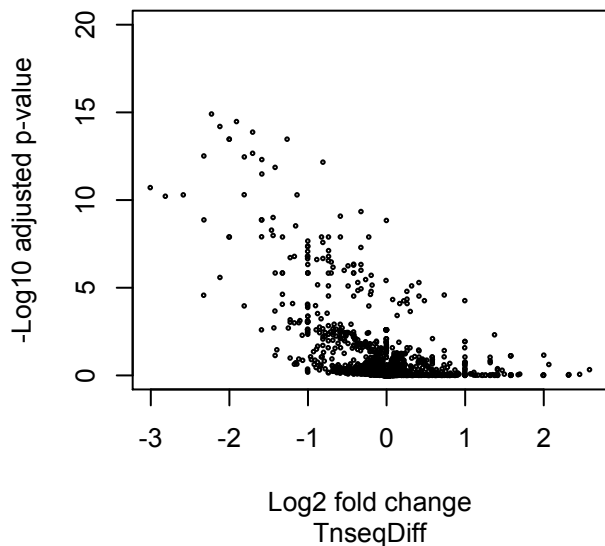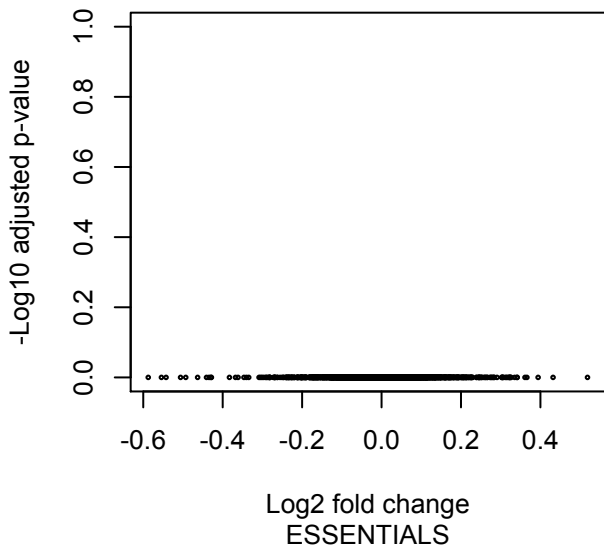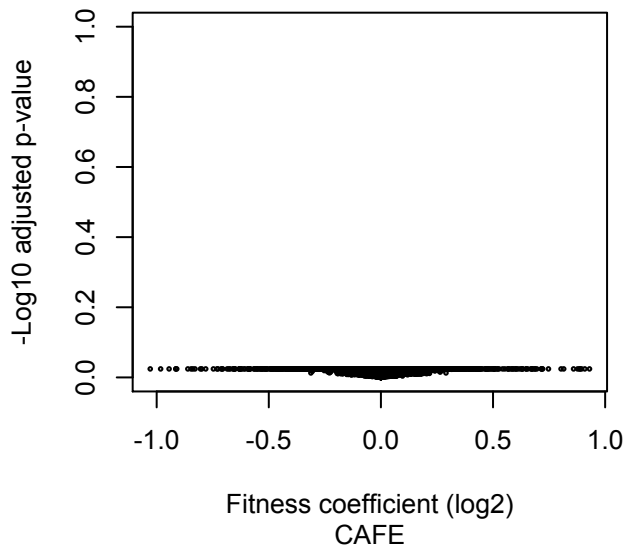

Supplement: btaa1086_Supplementary_Data [file btaa1086_supplementary_data.zip › Fig_S4.pdf]

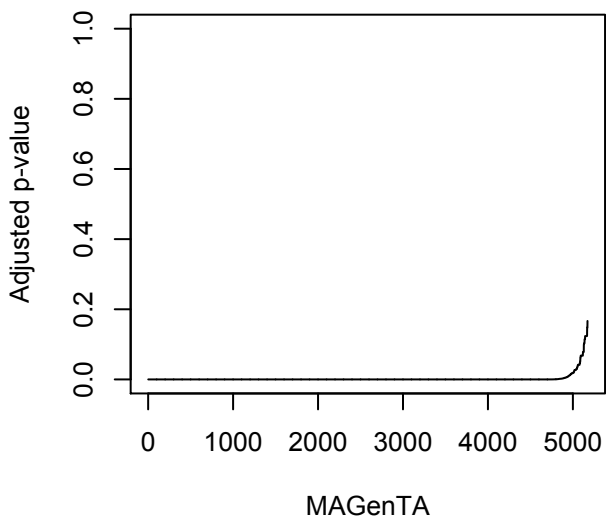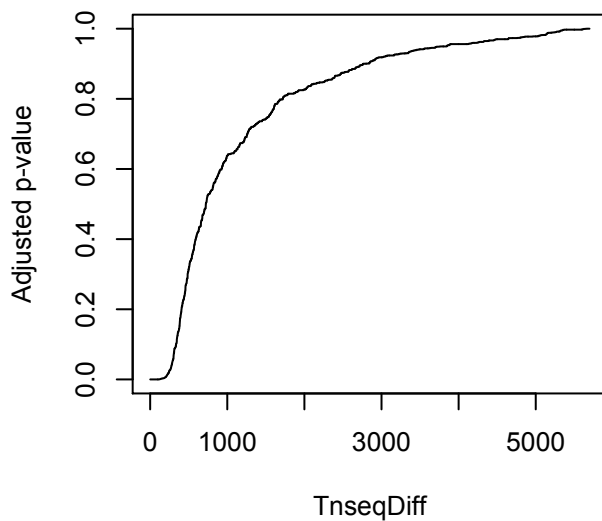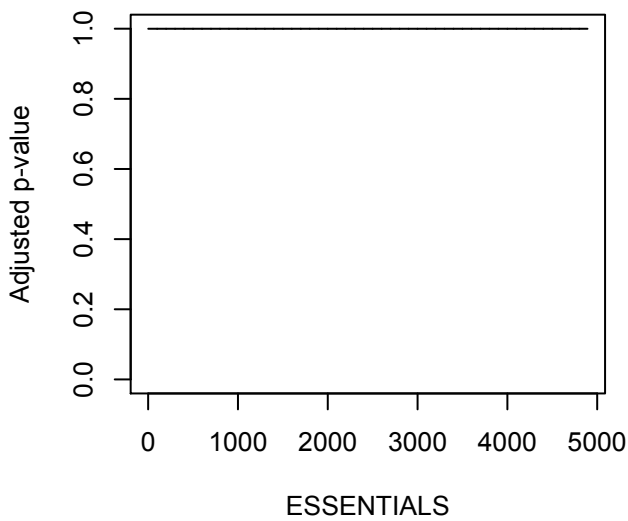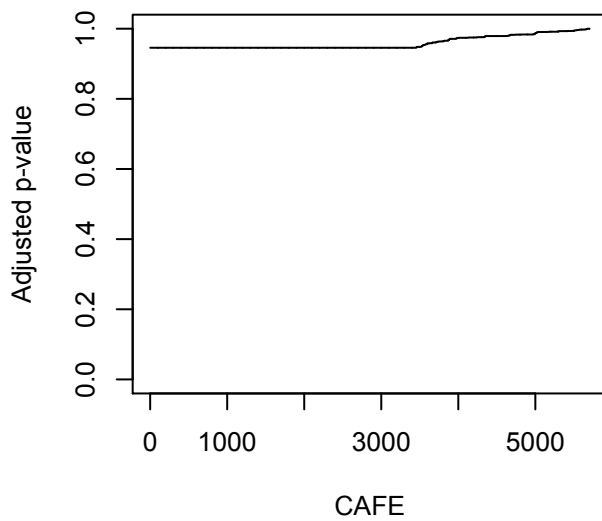

Supplement: btaa1086_Supplementary_Data [file btaa1086_supplementary_data.zip › Fig_S5.pdf]

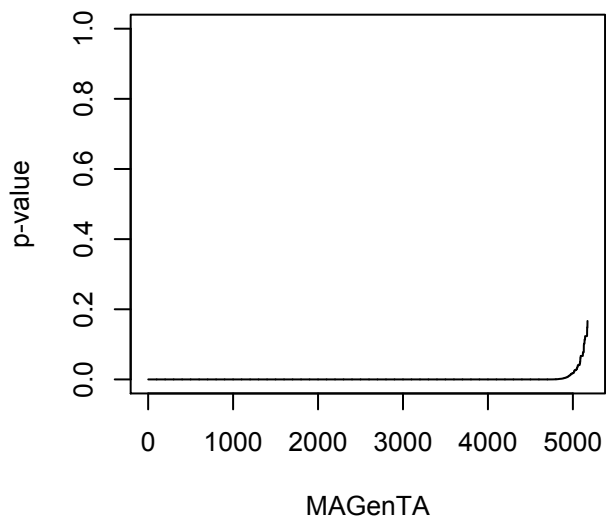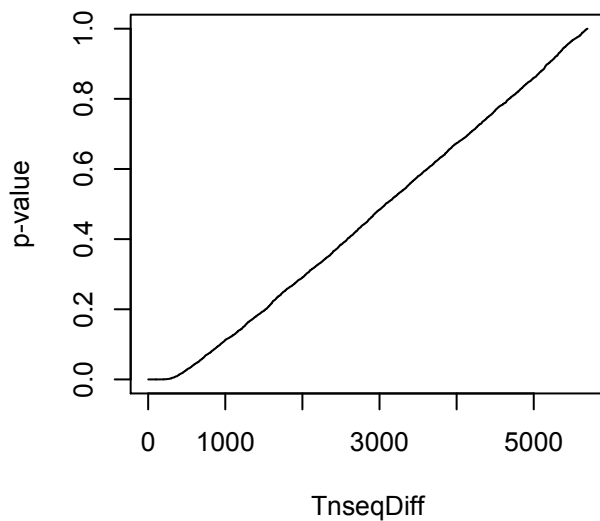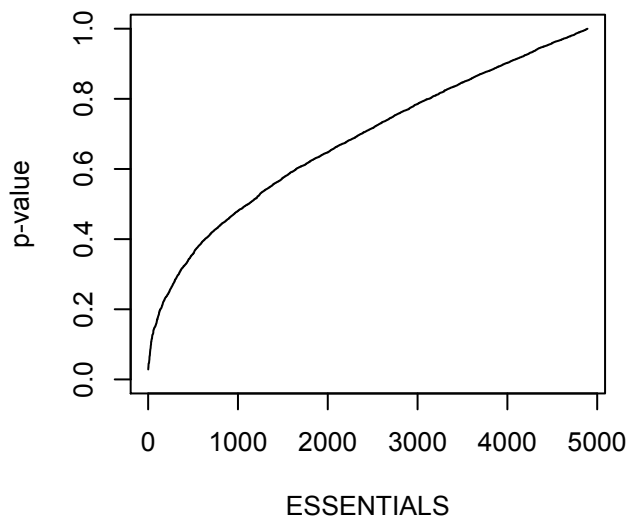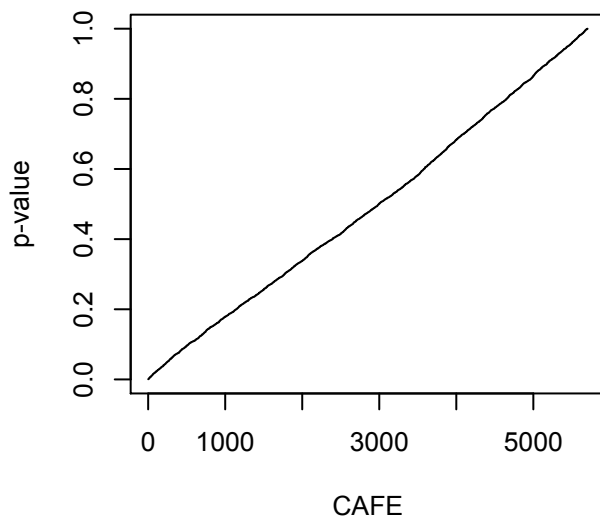

Supplement: btaa1086_Supplementary_Data [file btaa1086_supplementary_data.zip › Fig_S6.pdf]
